# Examination of the molecular control of ruminal epithelial function in response to dietary restriction and subsequent compensatory growth in cattle

**DOI:** 10.1186/s40104-016-0114-8

**Published:** 2016-09-15

**Authors:** Emma O’Shea, Sinéad M. Waters, Kate Keogh, Alan K. Kelly, David A. Kenny

**Affiliations:** 1 School of Agriculture and Food Science, University College Dublin, Belfield, Dublin, 4 Ireland; 2 Animal and Bioscience Research Department, Animal & Grassland Research and Innovation Centre, Teagasc Grange, Dunsany, Co. Meath, Ireland; 3 UCD Earth Institute, University College Dublin, Belfield, Dublin, 4 Ireland

**Keywords:** Beef cattle, Compensatory growth, Feed efficiency, Nutrient restriction, Rumen epithelium

## Abstract

**Background:**

The objective of this study was to investigate the effect of dietary restriction and subsequent compensatory growth on the relative expression of genes involved in volatile fatty acid transport, metabolism and cell proliferation in ruminal epithelial tissue of beef cattle. Sixty Holstein Friesian bulls (mean liveweight 370 ± 35 kg; mean age 479 ± 15 d) were assigned to one of two groups: (i) restricted feed allowance (RES; *n* = 30) for 125 d (Period 1) followed by *ad libitum* access to feed for 55 d (Period 2) or (ii) *ad libitum* access to feed throughout (ADLIB; *n* = 30). Target growth rate for RES was 0.6 kg/d during Period 1. At the end of each dietary period, 15 animals from each treatment group were slaughtered and ruminal epithelial tissue and liquid digesta harvested from the ventral sac of the rumen. Real-time qPCR was used to quantify mRNA transcripts of 26 genes associated with ruminal epithelial function. Volatile fatty acid analysis of rumen fluid from individual animals was conducted using gas chromatography.

**Results:**

Diet × period interactions were evident for genes involved in ketogenesis (*BDH2*, *P* = 0.017), pyruvate metabolism (*LDHa*, *P* = 0.048; *PDHA1*, *P* = 0.015) and cellular transport and structure (*DSG1*, *P* = 0.019; *CACT*, *P* = 0.027). Ruminal concentrations of propionic acid (*P* = 0.018) and n-valeric acid (*P* = 0.029) were lower in RES animals, compared with ADLIB, throughout the experiment. There was also a strong tendency (*P* = 0.064) toward a diet × period interaction for n-butyric with higher concentrations in RES animals, compared with ADLIB, during Period 1.

**Conclusions:**

These data suggest that following nutrient restriction, the structural integrity of the rumen wall is compromised and there is upregulation of genes involved in the production of ketone bodies and breakdown of pyruvate for cellular energy. These results provide an insight into the potential molecular mechanisms regulating ruminal epithelial absorptive metabolism and growth following nutrient restriction and subsequent compensatory growth.

## Background

Compensatory growth is an accelerated growth rate, upon re-alimentation, typically observed following a period of under-nutrition, to facilitate an animal in reaching its genetically pre-determined growth potential [18]. The compensatory growth phenomenon has traditionally been exploited by beef producers to reduce the overwintering feed costs of cattle production [2, 36]. Additionally, during compensatory growth, animals also exhibit enhanced feed efficiency [22], which can lead, not only to improved profitability but also to a reduction in ruminal methane emissions [6, 13] therefore reducing the carbon footprint of beef production. However, although the compensatory growth phenomenon is widely utilised throughout the world, there is a dearth of knowledge in relation to the biological control governing the expression of the trait.

Up to 75 % of a ruminants metabolizable energy supply is provided through volatile fatty acids (VFAs), which are generated from the ruminal fermentation of ingested plant material. Indeed it is estimated that 65 % of overall digestion occurs in the rumen alone [53], indicating the central importance of ruminal function to overall animal feed utilisation and efficiency. Diet composition has previously been shown to affect the absorptive metabolism in ruminal tissue of both sheep [7, 8, 52] and cattle [21, 39]. Additionally a period of feed restriction in cattle has been shown to increase total digestive tract digestibility [1]. Furthermore, the rumen has been shown to be one of the most responsive tissues to both dietary restriction and subsequent re-alimentation [22, 41, 57]. However, potential effects of compensatory growth following dietary restriction and subsequent re-alimentation, on ruminal epithelial function have not been assessed, to date. We hypothesized that in animals undergoing compensatory growth there would be an up-regulation of the relative expression of genes involved in VFA absorption and metabolism, as well as genes underlying growth and proliferation of the ruminal epithelium. We were also interested in investigating the possibility that the structural integrity of the rumen epithelium itself is altered during nutrient restriction. As such, the aim of this study was to examine the effect of dietary restriction and subsequent re-alimentation induced compensatory growth, on the expression of 26 genes involved in VFA transport, metabolism, growth and cellular structure in the ruminal epithelium of Holstein Friesian bulls. Animals were allowed a re-alimentation period of 55 d in order to capture the greatest increment of compensatory growth, as previously described by Hornick et al. [18].

## Methods

All procedures involving animals were approved by the University College Dublin Animal Research Ethics Committee and licensed by the Irish Department of Health and Children in accordance with the European Community Directive 86/609/EC.

### Animal model

This experiment was conducted as part of a larger study designed to examine the physiological and molecular control of compensatory growth in growing beef cattle [22]. Animals were managed on the same farm from two weeks of age prior to being transferred to Teagasc Grange Beef Research Centre, Dunsany, Co. Meath, Ireland. Sixty purebred Holstein Friesian bulls (mean liveweight 370 ± 35 kg; mean age 479 ± 15 d) were blocked on the basis of live weight, age and sire and were subsequently assigned within block to one of two dietary regimens (i) restricted feed allowance for 125 d (RES; *n* = 30) followed by *ad libitum* access to feed for a further 55 d (RES; *n* = 15) or (ii) *ad libitum* access to feed throughout the trial (ADLIB; *n* = 30). In order to acclimatise the animals to their environment and reduce any latent influence of previous environments, all animals were subjected to a 3 mon common feeding period of *ad libitum* grass silage plus 2 kg of concentrate per head per day prior to commencing the experiment. The first 125 d of the trial was denoted as Period 1 and the subsequent 55 d as Period 2.

All animals were offered the same diet consisting of 70:30 concentrate:forage (grass silage) throughout the entire trial, with RES animals receiving a restricted ration compared to ADLIB animals. Further details of the diet employed are provided by Keogh et al. [22] RES animals were managed to grow at 0.6 kg /d, with ADLIB animals expected to gain in excess of 1.5 kg/d during Period 1. Following completion of Period 1, 15 animals from each treatment (RES and ADLIB) were slaughtered. Prior to the commencement of Period 2 the previously restricted animals (RES) were allowed a 15 d transition period in order to build up to an *ad libitum* feed intake. This transition period was implemented to allow animals to acclimatise to a higher plane of nutrition while preventing the development of intestinal disorders, such as acidosis. All remaining bulls (*n* = 30) were then offered the control diet *ad libitum* for a further 40 d before slaughter. All animals were slaughtered in an EU licensed abattoir (Euro Farm Foods Ltd, Cooksgrove, Duleek, Co. Meath, Ireland). Slaughter order was randomized to account for potential confounding effects on treatment outcomes.

### Sample collection at slaughter

Tissue samples were excised post-mortem from the ventral sac of the rumen within 40 min of slaughter [26]. All instruments used for tissue collection were sterilized and treated with RNaseZap (Ambion, Applera Ireland, Dublin, Ireland) prior to use. Rumen papillae were harvested directly using a scissors. Samples were washed thoroughly with sterile, RNase free, phosphate buffered saline and subsequently snap frozen in liquid nitrogen before being stored at -80 °C.

Ruminal digesta was sampled from five different points within the rumen of each bull at slaughter, including the dorsal and ventral sacs. Rumen digesta was strained using cheese cloth, isolating the liquid fraction for VFA analysis. Rumen fluid samples were subsequently decanted to the appropriate vials using a graduated Gilsen pipette in order to facilitate appropriate digesta:preservative volumes. 20 mL samples were preserved with 0.5 mL of 9 mol/L sulphuric acid and stored at -20 °C for subsequent VFA analysis.

### VFA analysis

The concentration of VFAs (acetic, propionic, iso-butyric, n-butyric, isovaleric and n-valeric) collected at each slaughter time-point was measured in ruminal fluid using an automated gas chromatograph (Shimadzu Gas Chromatography GC-8A, Shimadzu Corporation, Kyoto, Japan; Brotz and Schaefer, 1987).

### RNA extraction and purification

Total RNA was isolated from approximately 100 mg of frozen rumen papillae tissue using TRIzol reagent and chloroform (Sigma-Aldrich Ireland, Dublin, Ireland). Tissue samples were homogenised using a rotor-stator homogenizing tissue lyser (Qiagen, UK), following which the RNA was precipitated using isopropanol. Samples were then purified using an RNeasy Plus Mini Kit (Qiagen, UK), according to the manufacturers instructions in order to remove any contaminating genomic DNA. The quantity of the RNA isolated was determined by measuring the absorbance at 260 nm using a Nanodrop spectrophotometer ND-1000 (Nanodrop Technologies, DE, USA). RNA quality was assessed on the Agilent Bioanalyser 2100 using the RNA 6000 Nano Lab Chip kit (Agilent Technologies Ireland Ltd., Dublin, Ireland). RNA quality was also verified by ensuring all RNA samples had an absorbance (A260/280) of between 1.8 and 2. RNA samples with 28S/18S ratios ranging from 1.8 – 2.0 and RNA integrity number of between 8 and 10 were deemed to be of high quality.

### cDNA synthesis

Total RNA (2 μg) was reverse transcribed into cDNA using a High Capacity cDNA Reverse Transcription Kit (Applied Biosystems, Foster City, CA, USA) using the Multiscribe™ reverse transcriptase according to manufacturers instructions. Samples were stored at -20 °C for subsequent analysis.

### Primer design and reference gene selection

Genes involved in the following processes were investigated in the current study: growth and structure, VFA metabolism, cellular transport proteins, ketogenesis and pyruvate metabolism. Gene specific primers (*n* = 26) used in this study were previously employed in studies of Penner et al. [39], Wang and Jiang [54] and Steele et al. [49] or specifically designed for use in the current study. Primer3 (http://frodo.wi.mit.edu/primer3/) and Primer BLAST (http://www.ncbi.nlm.nih.gov/tools/primer-blast/) software was utilized to design primers [24, 51]. Primer specificity was established using the Basic Local Alignment Search Tool (BLAST) from the National Centre for Biotechnology Information (http://www.ncbi.nlm.nih.gov/BLAST/). All primers targeting reference and candidate genes were obtained from a commercial supplier (Sigma-Aldrich Ireland, Dublin, Ireland). Details of primer sets used in this study are listed in Table 1. All amplified PCR products were sequenced to verify their identity (Macrogen Europe, Meibergdreef 39, 1105AZ Amsterdam, The Netherlands) and all amplicons were confirmed 100 % homologous to their target sequence.

**Table 1:** Sequences of oligonucleotide primers used for qRT-PCR

Gene name^a^	Accession number	Primer sequences	Amplicon size, bp
Endogenous control			
*RPLP0*	NM_001012682	F: AGGGCGTCCGCAATGTT	54
		R: CGACGGTTGGGTAACCAATC	
*GAPDH*	NM_001034034	F: GATTGTCAGCAATGCCTCCT	135
		R: CCATCCACAGTCTTCTGGGT	
*ATP5B*	NM_175796	F: CCCTCAAGGAGACCATCAAA	184
		R: GGACACCATGGAGGATGAGT	
*HPRT1*	NM_001034035	F: GCCGACCTGTTGGATTACAT	205
		R: GCATTGTCTTCCCAGTGTCA	
*B2M*	NM_173893	F: AGCGTCCTCCAAAGATTCAA	156
		R: ACAGGTCTGACTGCTCCGAT	
Cellular structure and growth			
*CCND1*	NM_001046273	F: GCACTTCCTCTCCAAGATGC	204
		R: GTCAGGCGGTGATAGGAGAG	
*CCND2*	NM_001076372	F: CCAGACCTTCATCGCTCTGT	163
		R: GATCTTTGCCAGGAGATCCA	
*CCND3*	NM_001034709	F: TCCAAGCTGCGCGAGACTAC	178
		R: GAGAGAGCCGGTGCAGAATC	
*CCNE1*	XM_612960	F: TTGACAGGACTGTGAGAAGC	187
		R: TTCAGTACAGGCAGTGGCGA	
*CCNE2*	NM_001015665	F: CTGCATTCTGAGTTGGAACC	229
		R: CTTGGAGCTTAGGAGCGTAG	
*CDKN1A*	NM_001098958	F: GCAGACCAGCATGACAGATT	205
		R: GTATGTACAAGAGGAGGCGT	
*CDKN2A*	XM_868375	F:GTGCGCCGGTTCTTGATTAC	105
		R: CCCATCATCATCACCCGCTG	
*CDKN2B*	NM_001075894	F: GCGGTGGATTATCCTGGACA	210
		R: CATCATCATCACCTGGATCG	
*DSG1*	NM_174045.1	F: AGACAGAGAGCAATATGGCCAGT	121
		R: TTCACACTCTGCTGACATACCATCT	
VFA activation			
*ACS*	DQ489534	F: GCTCTCACTGAGGAGCTCAAGAA	64
		R: AATCCGGTGTGGCAATGG	
*ACSS1*	BC114698.1	F: CCGATCAGGTCCTGGTAGTGA	200
		R: GAGCCATCACTTGGCACCTC	
*PCCA*	BC123876	F: AGAATGGAAGATGCCCTGGAT	70
		R: CCTCTCGAAGCAATGCGATAT	
Transport Proteins			
*CACT*	NM_001077936.2	F: TCACGCTCATGCGAGATGTT	94
		R: TTGACGCTCTTTCCCTCTGG	
*NHE1*	NM_174833.2	F: CCGTCACTGTGGTCCTGTAT	88
		R: CTCAGGAAGCCGAGGATGAT	
*NHE2*	XM_604493	F: TGCTCATCATGGTGGGACTT	83
		R: CACATCAGTCTTCATGGCCG	
*NHE3*	AJ131764.1	F: AGGTGCAGCTGGAGATCAT	102
		R: GACCTTGTTCTCGTCCAGGA	
Ketogenesis			
*ACAT*	NM_001034319.1	F: AATAGGCAGTGTTCGTCGGG	102
		R: CATGGACTCCACCCCACAAG	
*HMGCL*	NM_001075132	F: TGCAGATGGGAGTGAGTGTCA	73
		R: GACGCCCCCTGTGCATAG	
*BDH1*	NM_001034600	F: GACTGCCACCACTCCCTACAC	59
		R: TCCGCAGCCACCAGTAGTAGT	
*BDH2*	NM_001034488	F: CTGAAGAGGCACTGAGCGAT	98
		R: ATCAGAGGCCAAGTACACGC	
*HMGCS1*	AY581197	F: AGGATACTCATCACTTGGCCAACT	67
		R: CATGTTCCTTCGAAGAGGGAAT	
*HMGCS2*	NM_001045883	F: TCTGGCCCATCACTCTGCC	126
		R: AGTGGGGAGCCTGGAGAAGC	
Pyruvate Metabolism			
*LDHa*	BC146210	F: GGCAAAGACTATAATGTGACAGCAA	60
		R: ACGTGCCCCAGCTGTGA	
*LDHb*	BC142006	F: CCAACCCAGTGGATATTCTCACA	66
		R: TCACACGGTGCTTGGGTAATC	
*PC*	NM_177946	F: CTCCCACCATCTGTCCTTTCC	72
		R: TTTATTTGGGCAGGAGATGAATACG	
*PDHA1*	NM_001101046	F: CAGTTTGCTACTGCTGATCCTGAA	72
		R: AGGTGGATCGTTGCAGTAAATGT	

^a^ *RPLP0* ribosomal protein large P0, *GAPDH* glyceraldehyde 3-phosphate dehydrogenase, *ATP5B* ATP synthase subunit β, *HPRT1* hypoxanthine phosphoribosyltransferase 1, *B2M* β2 microglobulin, *CCND1* cyclin D1, *CCND2* cyclin D2, *CCND3* cyclin D3, *CCNE1* cyclin E1, *CCNE2* cyclin E2, *CDKN1A* cyclin dependant kinase inhibitor 1A, *CDKN2A* cyclin dependant kinase inhibitor 2A, *CDKN2B* cyclin dependant kinase inhibitor 2B, *DSG1* desmoglein 1, *ACS* acetyl coA synthetase, *ACSS1* acyl coA synthetase short-chain family member 1, *PCCA* propionyl coA carboxylase α polypeptide, *CACT* solute carrier family 25, carnitine/acylcarnitine translocase member 20, *NHE1* NA^+^/H^+^ exchanger isoform 1, *NHE2* NA^+^/H^+^ exchanger isoform 2, *NHE3* NA^+^/H^+^ exchanger isoform 3, *ACAT* acetyl coA acyltransferase, *HMGCL* 3-Hydroxymethyl-3-methylglutaryl-coA lyase, hydroxymethylglutaricaacidura, *BDH1* 3-Hydroxybutyrate dehydrogenase, type 1, *BDH2* 3-Hydroxybutyrate dehydrogenase, type 2, *HMGCS1* 3-Hydroxy-3-methylglutaryl-coA synthase 1, *HMGCS2* 3-Hydroxy-3-methylglutaryl-coA synthase 2, *LDHa* lactate dehydrogenase isoform a, *LDHb* lactate dehydrogenase isoform b, *PC* pyruvate carboxylase, *PDHA1* pyruvate dehydrogenase lipoamide, α 1

To determine relative gene expression level of target genes, five suitable reference genes were tested across all samples using qRT-PCR *viz*. glyceraldehyde 3-phosphate dehydrogenase (*GAPDH*), ribosomal protein large P0 *(RPLP0)*, ATP synthase subunit β *(ATP5B)*, hypoxanthine phosphoribosyltransferase 1 *(HPRT1)* and β2 microglobulin *(B2M)*. In order to select stable reference genes, reference gene expression data were analysed using GeNorm (GenEx 5.2.1.3, MultiD Analyses AB, Gothenburg, Sweden). GeNorm is a model-based approach software that measures the overall stability of the tested reference genes by calculating the intra-and intergroup variation and combining both coefficients to give a stability value (M value). A lower M value implies a higher stability in gene expression across all samples. An M value of 1.5 is specified as the default minimum coefficient by the GeNorm programme. In the current study, all reference genes tested displayed M values lower than 1.5.

### qRT-PCR

Following reverse transcription, cDNA quantity was determined and standardised to the required concentration for qRT-PCR. Triplicate 20 μL reactions were carried out in 96-well optical reaction plates (Applied Biosystems, Warrington, UK), containing 2 μL cDNA, 10 μL Fast SYBR Green PCR Master Mix (Applied Biosystems, Warrington, UK), 7 μL nuclease-free H_2_O, and 1 μL forward and reverse primers (250-1,000 nmol/L per primer). Assays were performed using the ABI 7500 Fast qRT-PCR System (Applied Biosystems, Warrington, UK) with the following cycling parameters; 95 °C for 20 s and 40 cycles of 95 °C for 3 s, 60 °C for 30s followed by amplicon dissociation (95 °C for 15 s, 60 °C for 1 min, 95 °C for 15 s and 60 °C for 15 s). Amplification efficiencies were determined for all candidate and reference genes using the formula E = 10^(1/slope), with the slope of the linear curve of cycle threshold (C_t_) values plotted against the log dilution [16]. Efficiency co-efficients of primers were estimated using serial dilutions, run as separate assays. Primers with efficiencies of between 90 and 110 % were deemed suitable. Interplate variation was accounted for by using an interplate calibrator, i.e., a common amplification was performed on the same sample across all plates. The software package GenEx 5.2.1.3. (MultiD Analyses AB, Gothenburg, Sweden) was used to correct for interplate variation, efficiency correction of the raw C_t_ values, normalisation to the reference genes and calculation of quantities relative to the maximum C_t_ value for each gene.

### Statistical analysis

Gene expression data were checked for normality using the UNIVARIATE procedure of Statistical Analysis Software (SAS version 9.3; SAS Institute Inc., Cary, NC). Where necessary, data were transformed using a Box-Cox transformation (PROC TRANSREG), by raising values to the power of λ. Data were subsequently analysed using mixed models ANOVA (PROC MIXED) with terms for diet and period as well as their interaction included as the main effects, with block included as a random effect in the statistical model employed. The choice of residual covariance structure was based on the magnitude of the Akaike Information Criterion (lowest is best) for models run under compound symmetry, unstructured, autoregressive, or Toeplitz variance-covariance structures. The Tukey critical difference test was performed to determine the existence of statistical differences between treatment LS mean values. Statistical significance was declared at *P* < 0.05 and trends at *P* < 0.10.

## Results

### Animal performance

The animal performance data have been presented in detail by Keogh et al. [22]. Briefly, average daily gain (ADG) for Period 1 was 0.6 kg/d for RES animals and 1.9 kg/d for ADLIB animals. During re-alimentation (Period 2) an ADG of 2.5 kg/d and 1.4 kg/d was observed for RES and ADLIB groups, respectively. This resulted in differences in bodyweight of 161 and 84 kg between RES and ADLIB treatments at the end of Period 1 and 2, respectively. A treatment by period interaction (*P* < 0.01) was detected for the weight of the reticulo-rumen when empty [22]. The empty reticulo-rumen was lighter in RES animals following a period of dietary restriction at the end of Period 1, however, there was no difference in empty reticulo-rumen weights between treatment groups at the end of Period 2.

### VFA analysis

The effects of diet and period on molar proportions of individual VFAs in the rumen fluid are outlined in Table 2. An effect of diet was observed on the proportions of propionic acid (*P* = 0.018) and n-valeric acid (*P* = 0.029) with reduced proportions in RES animals, compared with ADLIB, throughout the experiment. We also observed a tendency (*P* = 0.064) toward a diet × period interaction for the proportion of n-butyric acid with greater proportions in RES animals, compared with ADLIB, during Period 1, while the inverse was observed at the end of Period 2. No effect of diet was observed for concentrations of acetic acid, iso-butyric or iso-valeric acid (*P* > 0.05). Period effects were witnessed for the concentration of a number of VFA. Acetic (*P* = 0.024), propionic (*P* < 0.001), (*P* = 0.033) and n-valeric acid (*P* < 0.001) were all higher during Period 2 compared with Period 1 while the concentration of iso-butyric acid (*P* < 0.001) and iso-valeric acid (*P* = 0.029) was lower in Period 2 than in Period 1. Total VFA content of the rumen fluid was not affected by diet (*P* > 0.05) but was higher in Period 2, compared with Period 1 (*P* = 0.001), consistent with the overall increase in DMI in both treatment groups [22].

**Table 2:** Effect of diet (D) and period (P) on volatile fatty acid (VFA) levels^a^ in rumen fluid

	RES		ADLIB			Significance		
	Period 1	Period 2	Period 1	Period 2	SEM^b^	D	*P*	D*P^c^
Molar Proportions, mmol/L VFA								
Acetic Acid	60.9	67.79	52.14	77.57	6.875	0.942	0.024	0.186
Propionic Acid	13.98	28.44	20.1	34.75	2.506	0.018	<0.001	0.968
N-Butyric Acid	10.5	11.09	6.02	13.84	1.895	0.653	0.033	0.064
Iso-Butyric Acid	1.84	0.15	1.1	0.54	0.202	0.351	<0.001	0.562
N-Valeric Acid	0.88	2.18	1.38	2.87	0.261	0.029	<0.001	0.708
Iso-Valeric Acid	2.31	1.55	2.31	1.97	0.244	0.386	0.029	0.388
Total, mmol/L VFA	90.42	111.18	83.04	131.55	9.831	0.513	0.001	0.167
Acetate:Propionate Ratio	4.4	2.4	2.6	2.2				

^a^Ruminal fluid samples were taken from 5 different points within the rumen of each bull at slaughter and VFA levels were measured using an automated gas chromatograph

^b^SEM, standard error of the mean

^c^D*P, diet × period interaction

### Gene expression

The effects of diet and period on gene expression are outlined in Table 3. Diet × period interactions were evident for *DSG1* (*P* = 0.019); *CACT* (*P* = 0.027); *BDH2* (*P* = 0.017); *LDHa* (*P* = 0.048) and *PDHA1* (*P* = 0.015). These were manifested as follows. Expression of both *DSG1* and *CACT* was lower in RES animals compared to ADLIB at the end of Period 1, and subsequently greater in RES at the end of Period 2. mRNA expression of *BDH2*, *LDHa*, and *PDHA1* was greater in RES at the end of Period 1, with no difference observed between treatment groups at the end of Period 2. Additionally, there was a tendency (*P* = 0.053) towards a treatment × period interaction for *BDH1* which manifested as greater expression in RES, compared with ADLIB, animals during Period 1, but no difference between groups during Period 2. Lower expression levels across treatments in Period 1 compared with Period 2, resulted in period effects for the following genes: *CCND1* (*P* = 0.018); *CCND3* (*P* < 0.001); *CDKN2A* (*P* = 0.039); *PCCA* (*P* = 0.004); *NHE3* (*P* < 0.001); *ACAT* (*P* = 0.006); *HMGCS2* (*P* < 0.001); *LDHβ* (*P* = 0.002) and *PC* (*P* = 0.004). Whereas greater expression across treatments at the end of Period 1 compared with Period 2 led to a statistically significant effect of period on transcript abundance for *ACSS1* (*P* = 0.096); *NHE1* (*P* < 0.001) and *HMGCS1* (*P* < 0.001). The following genes were not affected (*P* > 0.05) by either dietary restriction or subsequent re-alimentation: *CCND2*, *CCNE1*, *CCNE2*, *CDKN1A*, *CDKN2B*, *ACS* and *NHE2*.

**Table 3:** Effect of diet (D) and period (P) on the relative expression^a^ of key genes in ruminal epithelial tissue

	RES		ADLIB			Significance		
Gene^b^	Period 1	Period 2	Period 1	Period 2	SEM^c^	D	*P*	D*P^d^
Growth and Structure								
*CCND1*	5.804	16.219	7.981	11.04	2.701	0.586	0.018	0.182
*CCND2*	6.544	20.787	13.521	13.724	4.054	0.618	0.183	0.192
*CCND3*	6.156	50.062	9.498	37.621	9.179	0.957	<0.001	0.396
*CCNE1*	20.302	43.776	31.736	30.536	8.105	0.913	0.185	0.137
*CCNE2*	58.975	106.52	86.475	79.928	27.07	0.913	0.948	0.325
*CDKN1A*	11.429	21.017	13.367	13.944	4.724	0.590	0.288	0.347
*CDKN2A*	5.6178	14.964	12.357	17.614	3.236	0.245	0.039	0.532
*CDKN2B*	13.851	23.052	14.281	12.594	5.554	0.372	0.503	0.334
*DSG1*	7.82	50.432	40.351	27.469	13.02	0.273	0.051	0.019
VFA activation								
*ACS*	2.72	2.359	2.12	2.588	0.155	0.826	0.103	0.733
*ACSS1*	2.579	2.047	2.703	2.079	0.338	0.818	0.096	0.893
*PCCA*	1.75	1.911	1.498	1.896	0.128	0.303	0.004	0.358
Transport proteins								
*CACT*	6.597	8.145	10.829	4.969	1.61	0.745	0.189	0.027
*NHE1*	17.983	7.76	15.672	8.039	1.238	0.418	<0.001	0.302
*NHE2*	3.455	2.964	2.94	3.197	0.355	0.695	0.745	0.300
*NHE3*	2.609	8.503	2.759	6.313	0.79	0.212	<0.001	0.148
Ketogenesis								
*ACAT*	12.153	31.857	12.779	20.89	4.802	0.289	0.006	0.235
*HMGCL*	2.313	5.241	3.169	5.171	0.42	0.356	<0.001	0.278
*BDH1*	3.971	1.67	3.29	1.99	0.251	0.476	<0.001	0.053
*BDH2*	3.491	2.803	2.1	2.931	0.304	0.045	0.815	0.017
*HMGCS1*	6.51	3.476	6.989	2.607	0.779	0.804	<0.001	0.392
*HMGCS2*	2.641	3.676	2.21	4.586	0.401	0.554	<0.001	0.103
Pyruvate metabolism								
*LDHa*	1.915	1.719	1.609	1.789	0.092	0.207	0.931	0.048
*LDHb*	1.749	2.151	1.685	2.074	0.121	0.564	0.002	0.959
*PC*	2.001	2.928	2.409	2.986	0.247	0.355	0.004	0.484
*PDHA1*	2.074	1.248	1.619	1.265	0.093	0.024	<0.001	0.015

^a^Gene expression values were normalized to the reference gene after adjustment for efficiencies and interplate variation and converted to values relative to the greatest cycle threshold (Ct) within each data set

^b^ *CCND1* cyclin D1, *CCND2* cyclin D2, *CCND3* cyclin D3, *CCNE1* cyclin E1, *CCNE2* cyclin E2, *CDKN1A* cyclin dependant kinase inhibitor 1A, *CDKN2A* cyclin dependant kinase inhibitor 2A, *CDKN2B* cyclin dependant kinase inhibitor 2B, *DSG1* desmoglein 1, *ACS* acetyl coA synthetase, *ACSS1* acyl coA synthetase short-chain family member 1, *PCCA* propionyl coA carboxylase α polypeptide, *CACT* solute carrier family 25, carnitine/acylcarnitine translocase member 20, *NHE1* NA^+^/H^+^ exchanger isoform 1, *NHE2* NA^+^/H^+^ exchanger isoform 2, *NHE3* NA^+^/H^+^ exchanger isoform 3, *ACAT* acetyl coA acyltransferase, *HMGCL* 3-Hydroxymethyl-3-methylglutaryl-coA lyase, hydroxymethylglutaricaacidura, *BDH1* 3-Hydroxybutyrate dehydrogenase, type 1, *BDH2* 3-Hydroxybutyrate dehydrogenase, type 2, *HMGCS1* 3-Hydroxy-3-methylglutaryl-coA synthase 1, *HMGCS2* 3-Hydroxy-3-methylglutaryl-coA synthase 2, *LDHa* lactate dehydrogenase isoform a, *LDHb* lactate dehydrogenase isoform b, *PC* pyruvate carboxylase, *PDHA1* pyruvate dehydrogenase lipoamide, α 1

^c^SEM, standard error of the mean

^d^D*P, diet × period interaction

## Discussion

The rumen is central to digestion in ruminants, with approximately 65 % of overall digestion occurring within the organ [53] and characterised by the supply of metabolisable energy in the form of microbial derived VFA. Given the central role of this organ to digestion it is not surprising that the rumen is one of the most responsive tissues to both dietary restriction and subsequent re-alimentation. Indeed, this was observed in the animals utilised in the current study [22] as well as in other studies investigating compensatory growth in cattle [41, 57]. However, despite its central importance to animal performance, the effect of feed restriction and subsequent compensatory growth on ruminal epithelial function has not been previously examined. In the current study we hypothesised that a period of dietary restriction, followed by subsequent re-alimentation induced compensatory growth, would affect VFA absorption and metabolism as well as cellular structure and growth of ruminal epithelial tissue. This was investigated through an examination of the expression of key component genes involved in each of these processes. We focussed on the first two mon of re-alimentation as the greatest increment of compensatory growth is known to occur during this period [18].

### VFA concentration and activation

In ruminants, VFAs are synthesized within the rumen through intensive microbial degradation of ingested carbohydrates. These short chain fatty acids are of central importance as an energy supply for the ruminant, providing up to 75 % of energy requirements. Previous reports in the literature have demonstrated that a period of feed restriction can reduce VFA absorption across ruminal epithelium [1, 59]. In the current study the molar proportions of six individual VFAs in rumen fluid were analysed namely: acetic, propionic, iso-butyric, n-butyric, iso-valeric and n-valeric acids following a 125 d period of dietary restriction and also following a subsequent 55 d re-alimentation period. Following dietary restriction at the end of Period 1, propionic acid concentrations were lower in RES animals compared to ADLIB. This result sustained through to the end of Period 2, where concentrations remained lower in RES animals. Propionic acid serves as a primary precursor of gluconeogenesis in ruminants [15]. During feed restriction a lower proportion of propionic acid present in ruminal fluid could be expected, due to lower feed intake and slower passage rate. However, our data indicate that propionic acid proportions remained lower than the control animals, when previously diet restricted animals were returned to an *ad libitum* diet. Alterations in microbial activity in the rumen as a consequence of reduced substrate availability most likely contributed to this observed reduction in propionic acid production.

Concentrations of n-valeric acid followed a similar pattern to propionic acid across both periods. Condensation of propionic acid with a 2-carbon unit intermediate synthesizes n-valeric acid, therefore a lower availability of n-valeric acid may be a direct consequence of the aforementioned reduction in propionic acid production [40]. Similarly, Whitelaw et al. [56] also observed a decline in ruminal n-valeric acid concentrations following a period of dietary restriction.

Recently, Minuti et al. [35] suggested that changes in the expression of genes involved in immune function, cellular proliferation and integrity and transport within rumen epithelial tissue of dairy cows, may be due to dietary alterations and possibly driven by nutrient induced changes in microbes and microbial metabolism. Indeed our own group has recently shown dramatic differences in rumen bacteria and archaea in solid and liquid fractions of rumen digesta from the same animals utilised in the current study, using phylogenetic amplicon sequencing technology [33]. Specifically we found a large reduction in the abundance of an uncharacterised Succinovibrionaceae and an increase in *Methanobrevibacter gottschalkii* consistent with the observed increase in the acetate:propionate ratio of RES cattle.

Intraruminal administration of acetate, propionate and butyrate have been shown to stimulate the growth and functional maturation of the rumen epithelium in young ruminants, with the effect of butyrate being most prominent toward rumen papillae proliferation [25, 42, 43, 47]. However, Wang and Jiang [54] observed that rumen fermentation does not directly stimulate rumen epithelial growth in cattle through increasing proliferation of rumen papillae epithelial cells. Shen et al. [46] reported elevated n-butyric acid concentrations in the rumen of goats fed energy-rich diets. Conversely, however, our data show that n-butyric acid proportions in the rumen tended to be greater when animals were undergoing a period of restricted growth. In addition to its proliferative effects on the rumen epithelium, n-butyric acid has been shown to be involved, in vivo, in the inhibition of ruminal apoptosis [34]. It is possible that greater proportions of n-butyric acid in RES animals could be involved in maintaining the ruminal papillae during a time of nutrient restriction, in order to maintain the efficiency of VFA absorption through increasing the surface area of rumen papillae. Although outside the scope of the current study, tissue morphology studies assessing rumen papillae growth and proliferation in relation to dietary restriction and subsequent re-alimentation are warranted. Although not reaching concentrations of ADLIB animals during re-alimentation, butyric acid concentrations did increase from Period 1 – Period 2 in restricted cattle, as the tissue increased in mass [22], potentially indicating the role of this acid in relation to rumen epithelial growth.

The relative gene expression of three enzymes involved in the process of VFA production were evaluated, namely; acyl-CoA synthetase short-chain family member 1 (*ACSS1*), acetyl CoA synthetase (*ACS*) and propionyl coenzyme A carboxylase, α polypeptide (*PCCA*). Both *ACSS1* and *ACS* encode enzymes involved in the conversion of acetate (a derivative of acetic acid) to acetyl coenzyme A (acetyl-coA), whilst the protein encoded by *PCCA* converts propionic acid to propionyl coenzyme A (propionyl-CoA). Following conversion, acetyl-CoA and propionyl-CoA can then be transported to the mitochondria through utilisation of the solute carrier family 25, carnitine/acylcarnitine translocase, member 20 (*CACT*). Although no effect of dietary restriction nor subsequent re-alimentation was observed for the expression of *ACSS1*, *ACS* or *PCCA*, *CACT* expression was affected. Lower expression of *CACT* was evident in RES animals at the end of Period 1, potentially indicating reduced transport of fatty acids into the mitochondria, and therefore reduced fatty acid catabolism. Down-regulation of *CACT* in RES animals may have prevented the shuttle-like action of carnitine from assisting transport of VFAs across the mitochondrial membrane, indicating that VFAs may build up within tissues. This possible reduction in the efficiency of fatty acid metabolism during nutrient restriction was partially restored during re-alimentation (Period 2). However, *CACT* expression in restricted animals upon re-alimentation did not reach the same level as that of control animals during Period 1. Despite this, had animals been allowed a longer re-alimentation period we may have observed full recovery of *CACT* transcript abundance.

### Cellular structure and growth

In the animals utilised in the current study, we previously observed greater reticulo-rumen weight for ADLIB animals at the end of Period 1, but not Period 2 [22], indicating full compensation of the organ during the first two months of re-alimentation. Wang and Jiang [54] previously observed an inhibitory effect of rumen fluid on proliferation in rumen papillae. More specifically butyrate has been shown to arrest cell cycle progression [12, 27, 28, 44, 55] and induce cyclin-dependent kinase inhibitor expression [4, 17, 32, 37, 48]. In the current study, given the greater concentration of butyrate recorded at the end of Period 1 in RES animals, the expression of cyclins and cyclin dependent kinase inhibitors were evaluated. However, neither nutrient restriction nor subsequent re-alimentation, had an effect on the expression of cyclin dependant kinase inhibitors (1A, 2A or 2B) or regulatory cyclins (D1, D2, D3, D4, E1 and E2) involved in the inhibition, or progression, respectively, of the cell cycle from the G1 phase to the S phase. We had hypothesized that accelerated growth typically observed during re-alimentation may be controlled, in part, by enhanced VFA absorption mediated by rumen papillae proliferation. However, the results of the current study showed no change in the transcription of genes involved in the cell cycle, indicating that this increase in organ size may be a result of other factors, such as cell hypertrophy or expansion of the reticulo-rumen organ. This was consistent with the increased DMI of the animals [22]. Had earlier tissue sampling been possible we may have indeed observed greater variation in ruminal epithelial gene expression profiles during re-alimentation.

Structural properties of rumen papillae may be altered due to differences in dietary intake. For example, Steele et al. [50] identified structural adaptations in the rumen epithelium of cows offered a high grain diet. Of particular interest, the authors of that study observed lower expression of *DSG1*, a desmoglein in response to the high concentration diet [50]. Furthermore, Steele et al. [50] also observed that expression of *DSG1* subsequently increased and remained elevated (compared with baseline values) following transition to a high forage diet [50]. In the current study, *DSG1* expression was lower in RES animals in Period 1, and subsequently greater in these animals at the end of the re-alimentation period. *DSG1* encodes an adhesion molecule involved in maintaining the structural integrity of epithelial cells including rumen epithelium. Lower expression of *DSG1* following dietary restriction could indicate a potential alteration of tight junctions between cells in the rumen epithelium. An alteration of the structure of the rumen wall may contribute to increased permeability or paracellular transport which may be an efficient mechanism of increased VFA absorption from the rumen in response to nutrient restriction. Using Cr-EDTA as a paracellular marker indicating gut tract barrier function, Zhang et al. [59] showed that the gut barrier was impaired following short-term feed restriction (5 d). Additionally, the authors of that study also demonstrated that tract barrier recovery time was affected by the severity of feed restriction [58]. During re-alimentation in Period 2, *DSG1* expression was subsequently greater in RES animals. This enhanced *DSG1* expression could indicate a restoration in the ruminal wall barrier function, which plays a role in regulating the permeability of molecules via paracellular pathways as well as bacterial translocation across the gut epithelium. This observed up-regulation of *DSG1* during re-alimentation indicates that the possible deterioration of the rumen wall during periods of nutrient deprivation could be a reversible process, with recovery following restoration of feed supply.

The expression of three ion transporters from a family of integral membrane protein transporters, known as the Na^+^/H^+^ exchangers (*NHE1, NHE2, NHE3*) was also evaluated in response to restricted feeding and subsequent re-alimentation. Previous in vitro studies have shown that rumen epithelium cells express high levels of NHE transporters [10, 11]. Moreover, enhanced absorption of VFAs is thought to be achieved through increasing the epithelial surface, which subsequently leads to an increase in the activity of ion exchangers [9, 30]. Additionally, active transport of Na^+^ has been shown to be stimulated by the presence of VFA across isolated rumen epithelium tissue mounted in Ussing chambers [5, 45]. However, in this study, no effect of diet was observed on the expression of three NHE transporters, suggesting that previously reported stimulation of Na + active transport by diet, was not the result of alterations in NHE transporter expression, but possibly due to greater presence of ATP for Na^+^/H^+^ exchanger function.

### Ketogenesis

When nutrient demand is high, fatty acid stores within body tissues are enzymatically broken down through β-oxidation to form acetyl-CoA. Under normal conditions, acetyl-CoA is further oxidized and subsequently enters the tricarboxylic (TCA) cycle. However, if activity in the TCA cycle is low due to low availability of intermediates, such as oxaloacetate, acetyl-CoA is used instead for the synthesis of ketone bodies *viz*. β-hydroxybutyrate (βHBA) [14]. This results in a rise in plasma βHBA in response to dietary restriction [23] and during early lactation in dairy cows [3] when nutrient demand is high. Plasma βHBA levels have also previously been shown to be elevated in heifers of poor feed efficiency [20]. In the current study we examined the expression of six genes involved in the pathway of ketogenesis; acetyl-coA acyltransferase 1 *(ACAT)*, 3-Hydroxymethyl-3-methylglutaryl-CoA lyase *(HMGCL)*, 3-Hydroxy-3-methylglutaryl-CoA synthase 1 *(HMGCS1)*, 3-Hydroxy-3-methylglutaryl-CoA synthase 2 *(HMGCS2)*, 3-Hydroxybutyrate dehydrogenase, type 1 *(BDH1)* and 3-Hydroxybutyrate dehydrogenase, type 2 *(BDH2*). Of the genes investigated, both *BDH1* and *BDH2* were affected by dietary restriction and subsequent re-alimentation, with greater expression observed for each gene in RES animals at the end of the dietary restriction period. This result suggests that during nutrient restriction, there may be greater emphasis on the production of βHBA from acetyl-CoA, potentially due to lower supply of intermediate substrates for the production of cellular energy through the TCA cycle. Indeed greater expression of *BDH1* has previously been associated with nutrition-induced ketosis in the liver of peri-parturient dairy cows [31].

### Pyruvate metabolism

Pyruvate is an important intermediate in key pathways of energy metabolism, thus the expression of four genes involved in pyruvate metabolism, namely pyruvate carboxylase (*PC*), pyruvate dehydrogenase lipoamide α 1 (*PDHA1*), lactate dehydrogenase isoform A (*LDHa*) and lactate dehydrogenase isoform B (*LDHb*) was investigated. Greater expression of *LDHa* was evident in RES animals at the end of Period 1, compared to ADLIB animals. *LDHa* is a cytoplasmic enzyme involved in the reversible catalysis of anaerobic glycolysis to produce lactate from pyruvate. Increased expression of *LDHa* during nutrient restriction suggests an increase in lactate production. Lactate production occurs when oxygen levels are low and can be necessary in order to regenerate NAD^+^, which is consumed in the synthesis of pyruvate from glucose, ensuring that energy production is maintained [19]. In addition to greater expression of *LDHa* at the end of Period 1, expression of *PDHA1* was also greater in RES animals at the same time. The pyruvate dehydrogenase complex is central to pyruvate metabolism through its role in the irreversible conversion of pyruvate to acetyl-CoA [38]. Previous reports in the literature have described a reduction in the expression of *PDHA1* in cattle fed a high compared with a low concentrate diet [39]. The authors of that study attributed the result to an increased butyrate supply from a high concentrate diet resulting in greater availability of acetyl-coA, thus potentially reducing the requirement for acetyl-CoA production by *PDHA1*. Conversely, our results suggest an increased reliance on *PDHA1* to produce acetyl-coA for the production of energy during nutrient restriction, possibly due to lower VFA, and therefore acetyl-coA supply from the diet. Similar to our own results, Lkhagvadorj et al. [29] observed greater expression of *PDHA1* in the-hepatic tissue of pigs following a period of dietary restriction compared to *ad libitum *controls.

## Conclusion

This is the first study to examine the effects of dietary restriction and subsequent re-alimentation induced compensatory growth on the transcript abundance of genes associated with the molecular functionality of ruminal epithelial tissue. The results of this study suggest that during dietary restriction the structural capacities of the rumen wall may be altered due to down-regulation of *DSG1* and the fatty acid transporter *CACT*. Additionally an up-regulation of genes involved in the production of ketone bodies and breakdown of pyruvate during dietary restriction, may have been necessary in order to maintain cellular energy requirements during restricted nutrient availability. Our data provide an insight into the potential molecular mechanisms regulating ruminal epithelial absorptive metabolism and growth following nutrient restriction and subsequent compensatory growth. Identifying key genes and pathways that contribute to enhanced feed efficiency in beef cattle and their implementation through genomically assisted breeding programmes could ultimately improve the economic and environmental sustainability of beef production.

## Abbreviations

ADG: Average daily gain
ADLIB: *Ad libitum*-fed treatment group
RES: Restricted-fed treatment group
SAS: Statistical analysis software
TCA: Tricarboxylic acid
VFA: Volatile fatty acid
βHBA: Beta-hydroxybutyrate
